# Murine Depression Model and its Potential Applications for Discovering Foods and Farm Products with Antidepressant-Like Effects

**DOI:** 10.3389/fnins.2016.00072

**Published:** 2016-03-01

**Authors:** Tatsuhiko Goto, Shozo Tomonaga, Tsuyoshi Okayama, Atsushi Toyoda

**Affiliations:** ^1^Department of Biological Production Science, College of Agriculture, Ibaraki UniversityAmi, Ibaraki, Japan; ^2^Department of Biological Production Science, Ibaraki University Cooperation between Agriculture and Medical ScienceAmi, Ibaraki, Japan; ^3^Graduate School of Agriculture, Kyoto UniversityKyoto, Japan; ^4^Department of Biological Production Science, United Graduate School of Agricultural Science, Tokyo University of Agriculture and TechnologyFuchu, Japan

**Keywords:** depression, food, behavior, metabolomics, social defeat stress, depth camera

## Abstract

Advanced societies face increased health problems related to various stresses. Chronic psychological stress is a major risk factor for psychiatric disorders such as depression. Although therapeutic agents reduce several symptoms of depression, most have side effects in a broad range of the population. Furthermore, some victims of depression do not show significant improvement with any drugs, so alternative approaches are needed. Good dietary habits may potentially reduce depressive symptoms, but there is little scientific evidence thus far. Murine depression models are useful to test nutritional approaches *in vivo*. Our model mice subjected to a subchronic mild social defeat stress (sCSDS) paradigm show several alterations in physiological parameters and social behavior. These stress-induced symptoms in sCSDS mice can be used as cues to identify antidepressant-like natural resources including foods and farm products. We previously discovered that sCSDS mice show more vulnerability to social stress by changing dietary condition. In addition, we developed a more objective system for analyzing mouse behavior using a 3D depth-sensing camera to understand relationships between diet and behavior. The combination of sCSDS mice with 3D behavioral analysis is a powerful method for screening ingredients in foods and farm products for antidepressant-like effects.

## Introduction

Advanced societies face increased health problems related to various stresses; chronic psychological stress in particular is a major risk factor for precipitating psychiatric disorders such as depression. A Global Burden of Disease study showed depression is the most disabling disorder worldwide (Whiteford et al., 2013). Since this issue will face the next generation of developing countries, it is imperative to find some solution. Therapeutic agents reduce several symptoms of depression, but most have several side effects in a broad population (Stevens et al., 2014; Galling et al., 2015). Moreover, some victims of depression do not show significant improvement with any drugs (treatment-resistant depression), so alternative approaches are needed (El-Hage et al., 2013). For prevention, it is more important to change dietary habits rather than resort to conventional treatments. Recently, interest has increased in Kampo (Watanabe et al., 2011) and functional foods (Arai, 1996). In fact, some reports indicate that Kampo (Ito et al., 2012; Hori et al., 2015) and functional ingredients (Tomonaga et al., 2008; Iio et al., 2012a) have antidepressant effects in animal models. This study further explores functional ingredients from natural sources for prevention and attenuation of symptoms of depression.

Chronic social defeat stress (CSDS) models of rats and mice are recognized as good animal models of depression. CSDS models establish social stress using male territorial aggression (Kudryavtseva et al., 1991; Miczek et al., 2008; Hammels et al., 2015). We previously studied CSDS rats and found that social stress induces alterations in the MAP kinase cascade, hypothalamic malonyl-CoA, peripheral leptin, digestive system, and behaviors (Iio et al., 2011, 2012b, 2014; Toyoda et al., 2015). Others have analyzed both central nervous system and peripheral tissues in relation to stress resilience using CSDS mice (Russo and Nestler, 2013; Hodes et al., 2014; Pfau and Russo, 2015). In addition, we have established a milder model of depression, subchronic mild social defeat stress (sCSDS; Goto et al., 2014, Goto and Toyoda, 2015) than CSDS (Krishnan et al., 2007). Since several models are required (Bartolomucci and Leopardi, 2009), sCSDS mice may provide insights about pathogenic mechanisms and preventive measures for depression.

The 3D behavioral analysis allows evaluation of more natural, realistic animal behavior. Although animals behave in 3D space and show several postures sterically, conventional animal monitoring systems have been primarily 2D video cameras. Recently, commercially available 3D depth-sensing cameras have been developed in the computer game industry, and can thus be affordably obtained (~$200/camera). Using 3D cameras, rats and mice have been monitored (Ou-Yang et al., 2011). Since behavioral scientists can derive depth information from animal behavior, the 3D camera enables discrimination of slight differences in 3D behavioral characteristics that have not been previously well detected.

In this article, we introduce features of our murine stress model and novel 3D monitoring system. We discuss the use of both for discovering foods and farm products providing antidepressant-like effects.

## Subchronic mild social defeat stress (sCSDS) model mouse

### Phenotypes of sCSDS (Figure 1A)

sCSDS mice are established by the method (Goto and Toyoda, 2015). Subject C57BL/6J mice are exposed to psychosocial stress from aggressive ICR mice for 10 consecutive days. During establishment, body weight gain, food intake, and water intake in sCSDS mice are significantly higher than those of non-stressed control mice (Goto et al., 2014). The sCSDS mice show increased body water content and social avoidance behavior after the stress. Moreover, nest-building behavior in sCSDS mice is significantly delayed compared to control mice (Otabi et al., 2016). In CSDS mice, social avoidance behavior has been widely reported (Tanaka et al., 2012; Russo and Nestler, 2013). Increased body weight gain has been reported in defeated mice (Goto et al., 2014) and lower ranking mice (Kim et al., 2015). Stress-induced polydipsia has been reported in both CSDS (Krishnan et al., 2007) and chronic mild stress (CMS) conditions (Gross and Pinhasov, 2016). Although, these features are supported by several studies, social stress-induced increases in body water content (Goto et al., 2014) and delays in nest-building behavior (Otabi et al., 2016) are, to our knowledge, unique findings.

**Figure 1:**
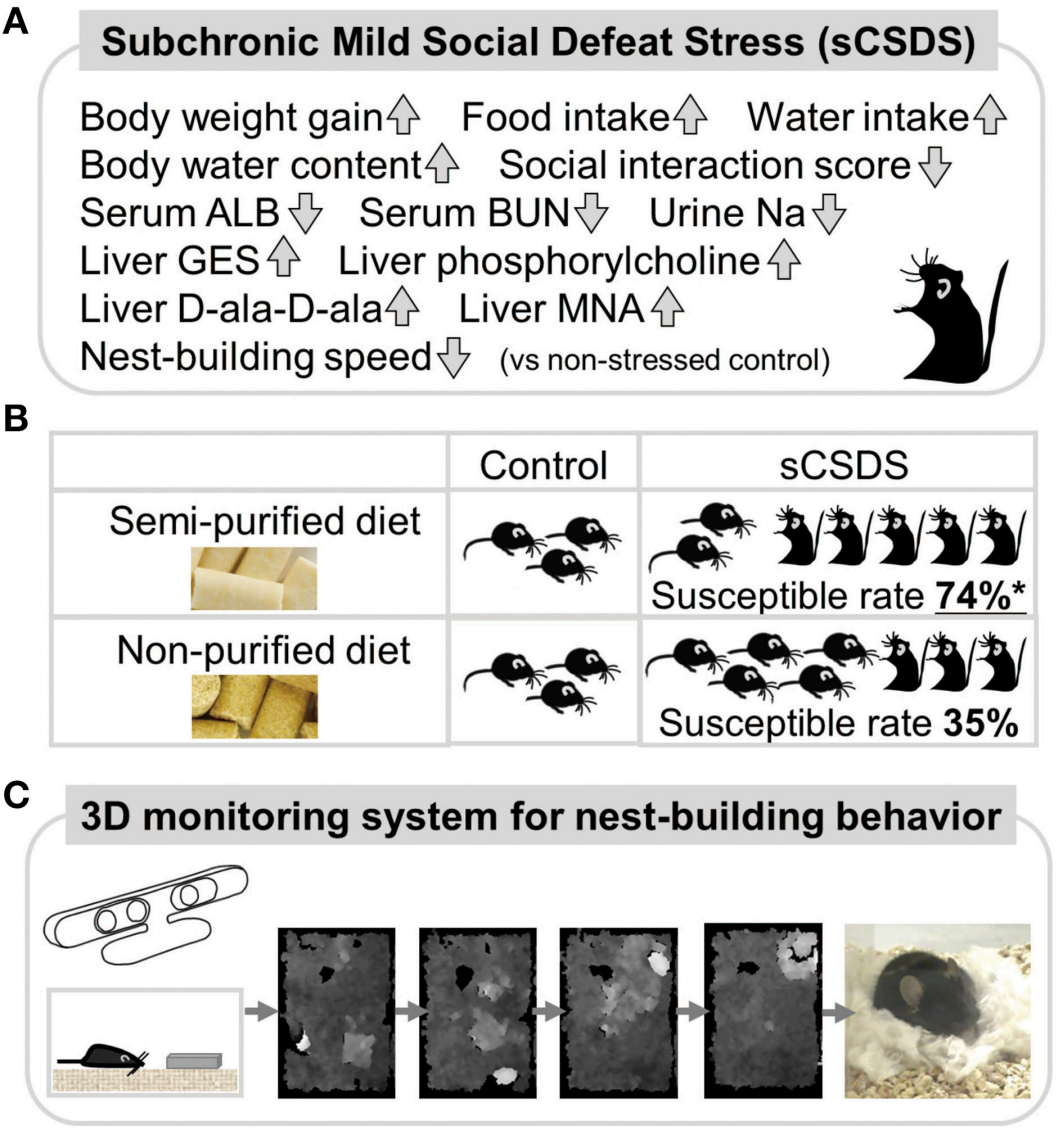
**Summary of a mouse model and 3D behavioral analysis.(A)** Social stress-induced symptoms in sCSDS mice. **(B)** Dietary quality influence stress-vulnerability behavior in sCSDS mice. sCSDS mice fed semi-purified diet indicate significantly higher susceptible rate than sCSDS mice fed non-purified diet. ^*^P < 0.05 (Goto et al., in press). **(C)** 3D monitoring system of nest-building behavior. Temporal changes of nesting behavior are clearly identified by depth images (higher regions are indicated by whiter color).

### Metabolomic analyses of sCSDS (Figure 1A)

To find key metabolites altered by social defeat stress, we tested the blood plasma/serum, urine, and liver. Biochemical assays for blood serum components revealed that sCSDS mice showed low levels of albumin (ALB) and blood urea nitrogen (BUN) just after stress (Goto et al., 2014). Low levels of sodium were found in the urine of sCSDS mice. Metabolomics revealed that four liver metabolites, taurocyamine (GES), phosphorylcholine, D-alanyl-D-alanine (D-ala-D-ala), and 1-methylnicotinamide (MNA), were significantly upregulated in sCSDS mice vs. control mice (Goto et al., 2015a).

### Food habits attenuate stress-induced symptoms in sCSDS (Figure 1B)

We established sCSDS mice under feeding conditions with two kinds of pellet food, a semi-purified and a non-purified diet. We confirmed that the increased body weight gain, food intake, and water intake of sCSDS mice during stress were common to both diets (Goto et al., in press). Interestingly, we found that the vulnerability of mice to social defeat stress was affected by diet quality. sCSDS mice fed a semi-purified diet were more susceptible than sCSDS mice fed a non-purified diet (Goto et al., in press). This may be due to changing gut environments, as gut microbiota and their metabolic products can affect animal brain function and behavior (Cryan and Dinan, 2012). Especially, commensal microbiota can influence the hypothalamus-pituitary-adrenal reaction to stress in mice (Sudo et al., 2004). And, gut microbiota can modulate brain development and modulation of the serotonergic system, which is directly related to mood, in the limbic system (Heijtz et al., 2011). Although the microflora of sCSDS mice have not been fully studied, intestinal flora will be influenced by both stress and food. Metabolomics with the sCSDS mice fed both diets hope to find some solutions to attenuate stress-induced behavior by changing the metabolic environments of peripheral tissues.

## A novel 3D behavioral testing method for innate behavior (Figure 1C)

Infrared 3D depth-sensing cameras have been developed intensely in the computer game industry. Both Microsoft Kinect (Microsoft Corp., USA) and Xtion PRO LIVE (ASUSTek Computer Inc., Taiwan) have become commercially available and affordable. The 3D depth-sensing camera has thus recently become available for use in rodent behavioral tests. The first reported behavioral analyses using a 3D camera focused on locomotion and pose in rats and mice (Ou-Yang et al., 2011). Matsumoto et al. constructed skeleton models of rats using four 3D cameras simultaneously and analyzed social and sexual interactions and novel object recognition behavior (Matsumoto et al., 2013, 2014). We monitored nest-building behavior in mice using a 3D camera (Okayama et al., 2015), and confirmed its effective utilization in a genetic study (Goto et al., 2015b). Nakamura et al. have developed a gait analysis system for mice using a 3D camera (Nakamura et al., 2015). Hong et al. succeeded in automating measurement of mouse social behaviors with machine learning algorithms from images captured simultaneously by a 3D camera and two 2D video cameras (Hong et al., 2015). Although conventional testing has been performed with 2D video cameras, future animal behavioral testing will evolve dramatically through use of 3D cameras.

Our 3D monitoring system focused on nesting behavior in mice, because the steric nest can be evaluated as a visible behavior. Deacon codified a standard method of nest-building behavior in mice using pressed cotton and rated nest quality on a scale of 1–5 (Deacon, 2006). By utilizing 3D depth-sensing cameras, we are able to conduct objective 3D evaluation of the final nest in one point evaluation (Okayama et al., 2015). In addition, we have analyzed an untapped behavioral characteristic, the construction process of the nest. Our 3D monitoring system could discriminate slight differences in temporal nesting behavior using 3D depth images and Deacon score 1-5 (Goto et al., 2015b). Since nest-building behavior is altered by social stress (Otabi et al., 2016), several stress models such as CSDS, CMS, and restraint stress should also be analyzed while nest-building in future.

## Strategy for finding antidepressants from natural ingredients (Figure 2)

Using sCSDS mice, we will investigate functional ingredients from natural sources that should attenuate stress-induced symptoms. Since sCSDS mice fed a semi-purified diet show more vulnerable behavior than mice fed a non-purified diet (Goto et al., in press), a change to a semi-purified diet as a base diet would be a good approach to identify functional ingredients enhancing stress resilience behavior. A semi-purified diet is preferred for nutritional research because the non-purified diet contains unknown raw materials and ingredients. Since the non-purified diet potentially improves stress vulnerability, it would be worthwhile to note differences between semi-purified and non-purified diets. Components including dietary fiber, resistant starch, and unavailable carbohydrates will be targets in future studies. These components are available to gut microbiota, resulting in a variety of species living in the gut. The brain-gut axis (Kelly et al., 2015) should be a main target for food-mediated approaches to finding antidepressants and preventing stress-related diseases by shifting food habits. In addition, sCSDS mice show stress-induced symptoms described above. By checking these indicators, the depression model can be useful for screening antidepressant effects of functional food and farm products.

**Figure 2:**
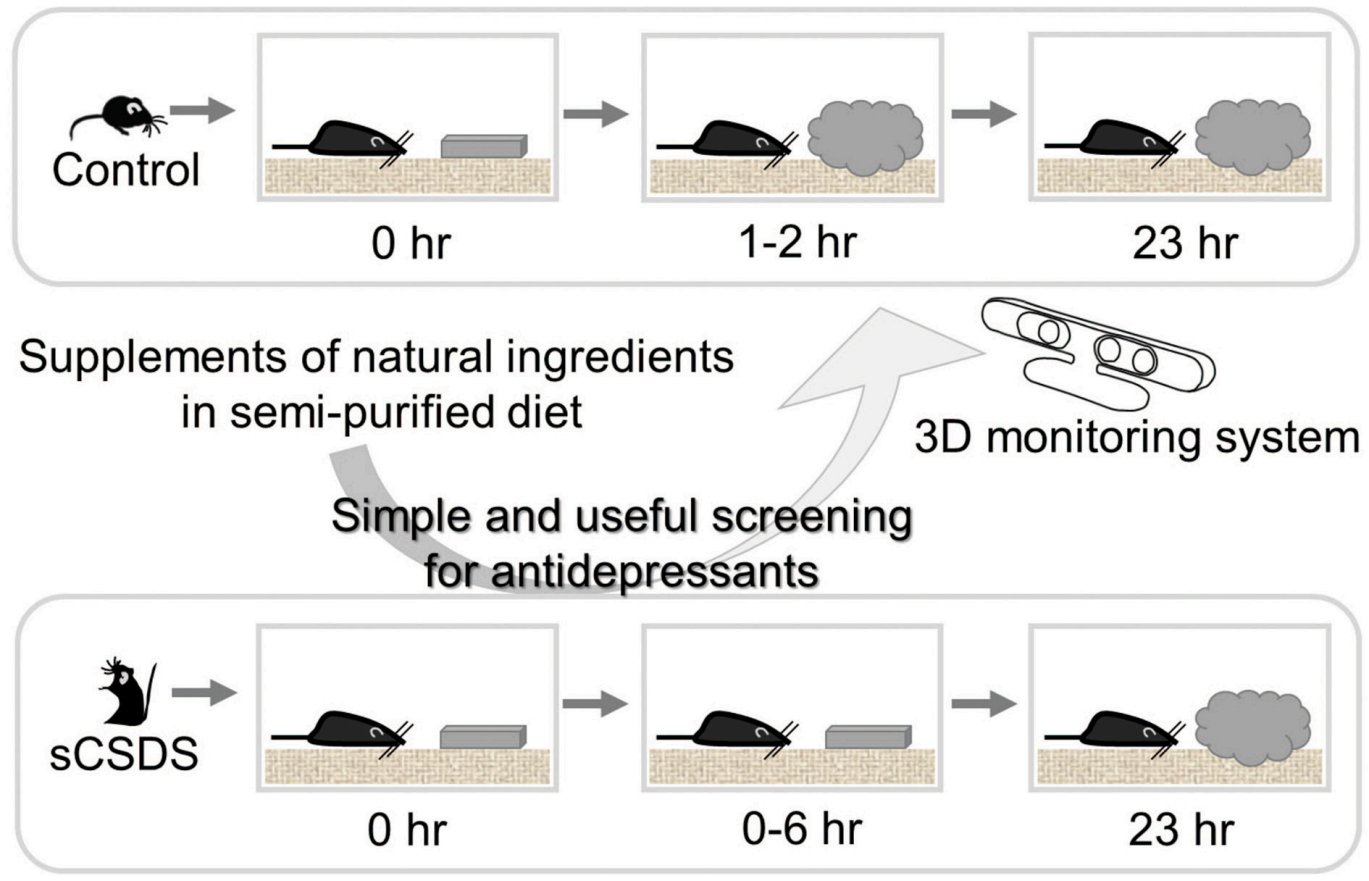
**Strategy for finding antidepressants from natural sources**. The combination of sCSDS mice with 3D monitoring system is a powerful method for screening antidepressants from natural ingredients. Natural ingredients which rescue retarded nest-building can be screened.

Our novel 3D monitoring system for nest-building enabled us to detect small differences among groups in different treatments and strains (Okayama et al., 2015; Goto et al., 2015b). Furthermore, we found simple and useful nesting behavioral test (Otabi et al., 2016). By using this combined method with sCSDS mice and 3D monitoring systems, we can discern slight differences in innate behavior and screen candidate supplements quickly. In future, automated behavioral analysis for nesting behavior will make this method easy for many researchers.

Toyoda and colleagues conducted research in the interdisciplinary field of agricultural-medical science (http://iucam-ibaraki.wix.com/iucam), exploring functional food and farm products for prevention and attenuation of psychiatric symptoms. Establishing animal models of depression, searching and screening supplemental resources, and applying engineering techniques will be essential in the big data era. Now that the fundamental platform has been established, we can screen ingredients from natural sources such as dairy products, fruits, and vegetables.

## Conclusions

In this article, we introduced our mouse model of depression and novel 3D evaluation system for nesting behavior. Good dietary habits should provide potential effects for reducing depressive symptoms and extending healthy life. The importance of food habits in overcoming stress can be shown by identifying antidepressant-like natural resources using sCSDS mice. The combination of sCSDS mice with 3D behavioral assays is a powerful method for screening the ingredients showing antidepressant-like effects in foods and farm products.

## Author contributions

TG and AT wrote overall manuscript. ST and TO wrote a part of the manuscript about metabolome and 3D sensor, respectively. TG, ST, TO, and AT have checked the manuscript entirely and agree with submission.

## Funding

This research was supported in part by an Ibaraki University Cooperation between Agriculture and Medical Science (IUCAM) (The MEXT, Japan) and the Council for Science, Technology and Innovation (CSTI) under the Cross-ministerial Strategic Innovation Promotion Program (SIP) “Technologies for creating next-generation agriculture, forestry, and fisheries” (Bio-oriented Technology Research Advancement Institution, NARO) (The Cabinet Office, Japan).

### Conflict of interest statement

The authors declare that the research was conducted in the absence of any commercial or financial relationships that could be construed as a potential conflict of interest.
